# Platelet-rich plasma in the management of Asherman's syndrome: An RCT

**DOI:** 10.18502/ijrm.v18i2.6423

**Published:** 2020-02-27

**Authors:** Atiyeh Javaheri, Katayoon Kianfar, Soheila Pourmasumi, Maryam Eftekhar

**Affiliations:** ^1^Department of Obstetrics and Gynecology, Shahid Sadoughi Hospital, Shahid Sadoughi University of Medical Sciences, Yazd, Iran.; ^2^Non-Communicable Diseases Research Center, Rafsanjan University of Medical Sciences, Rafsanjan, Iran.; ^3^Research and Clinical Center for Infertility, Yazd Reproductive Sciences Institute, Shahid Sadoughi University of Medical Sciences, Yazd, Iran.; ^4^Abortion Research Center, Yazd Reproductive Sciences Institute, Shahid Sadoughi University of Medical Sciences, Yazd, Iran.

**Keywords:** Asherman's syndrome, Platelet-rich plasma, Pregnancy rate.

## Abstract

**Background:**

Asherman's syndrome (AS) is a rare reproductive abnormality, resulting in endometrial collapse due to aggressive or recurrent endometritis and/or curettage.

**Objective:**

We aimed to assess the effectiveness of using platelet-rich plasma (PRP) to lower the recurrence rate of intrauterine adhesions (IUAs) following hysteroscopy.

**Materials and Methods:**

In this non-randomized clinical trial, women aged 20-45 years with AS diagnosed by sonohysterography, 3D sonography, hysteroscopy, or uterosalpingography between May 2018 and September 2018 were included. Participants (n = 30) were divided into case and control groups. Following hysteroscopic adhesiolysis, a Foley catheter was placed into the uterine cavity in all women. After two days, the catheter was removed, and 1-mL PRP was injected into the uterine cavity of women in the PRP (case) group, while the control received no PRP. All controls and subjects underwent diagnostic hysteroscopy 8-10 weeks following the intervention to assess the IUAs according to the American Society for Reproductive Medicine scoring system.

**Results:**

Our results did not reveal any significant difference in the menstrual pattern of either the control or test groups before or after treatment (p = 0.2). Moreover, the IUA stage in both studied groups before and after treatment was similar (p = 0.2). The duration of menstrual bleeding in both studied groups before and after treatment was also similar.

**Conclusion:**

PRP cannot change the menstrual pattern or development of postsurgical AS, as evaluated by follow-up hysteroscopy.

## 1. Introduction 

As a unique tissue in adult humans, the uterine endometrium bears the potential to sustain some changes such as shedding physiologically at certain intervals followed by regeneration at virtually monthly periods unless a female's reproductive system fails to function properly. cooperation of some tissues including endothelial, epithelial, and progenitor cells as well as fibroblasts and their reaction to local signaling from tissue microenvironment assists in successful implementation of this critical repairing process of tissues. The process subsumes proliferation of the cells, their migration, accompanied by lineage differentiation, and transdifferentiation via mesenchymal-to-epithelial transition. However, if this tightly regulated homeostatic balance is disturbed, the consequences are endometrial pathologies, poor pregnancy outcomes, and infertility (1).

One of the unusual gynecological disorders induced by the destruction of the endometrium and emerges from frequent or aggressive curettage and/or endometritis is Asherman's syndrome (AS) (2). Consequently, in many areas, a loss of functional endometrium occurs and is accompanied by obliteration of the uterine cavity, which results from intrauterine adhesions (IUAs) and induces infertility, recurrent pregnancy loss, hypomenorrhea, amenorrhea, placenta previa, and placenta accrete (3).

Management and therapeutic strategies involve hysteroscopic adhesiolysis and re-adhesion prevention using intrauterine devices, Foley's catheter, uterine balloon stent, or amnion grafts (4).

In order to help generate a healthy layer of the cells and hence support pregnancy, the AS management should, therefore, be oriented toward eliminating any adhesion and controlling their regeneration.

To restore endometrial function, some therapeutic attempts have been made including the application of exogenous estrogen, vaginal sildenafil citrate, aspirin, pentoxifylline and vitamin E; yet the outcomes need to be improved (4).

Comprising serum with a platelet concentration greater than 10 × 10${}^{5}$ platelets per cubic microliter, platelet-rich plasma (PRP) is a rich source of growth factors that has antigenic and mitogenic features and includes platelet-derived growth factor, epidermal growth factor, fibroblast growth factor, transforming growth factor-beta, and vascular endothelial growth factor (5).

The role these growth factors in modulating endometrial cell proliferation and differentiation has been identified. Several investigations to date have assessed the role of intrauterine instillation of autologous PRP in suboptimal endometrium, that is, to improve embryo transfer (ET) and vascularity (6-8). Hence, PRP is expected to exert beneficial effects on damaged endometrium. In addition, based on long-term clinical experience, the use of PRP is considered safe, and unlike bone marrow-derived stem cells (BMDSCs), it can be much more readily obtained (9).

The present study aimed to evaluate the impact of PRP use in reducing the recurrence of IUAs following their lysis by hysteroscopy.

## 2. Materials and Methods

In this non-randomized clinical trial, 30 women aged 20 to 45 years with AS diagnosed based on 3D sonography, sonohysterography, hysterosalpingography, or hysteroscopy from May 2018 to September 2018 were included. The women were referred to the Research and Clinical Center for Infertility, Yazd, Iran and were divided into two groups: case (PRP group) and control (n = 15/each) groups. Women who used anticoagulants or NSAIDs within at least 10 days prior to the procedure, those with any major disorders that might have compromised the ability of patients to provide consent, and those with active cervical or uterine infections were excluded. For those in the case group, 1 ml of PRP was injected into the uterine cavity, whereas for those in the control group, no injection was given.

**Figure 1:**
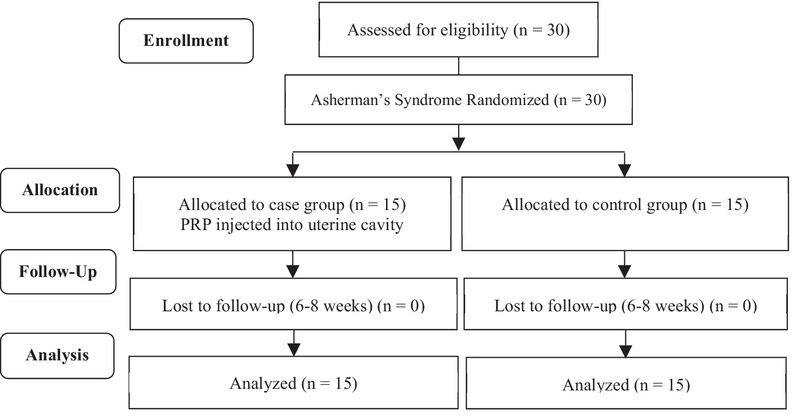
Consort flow diagram of study.

The demographic data, laboratory, and checkup results prior to and after treatment were recorded. The severity of IUA was appraised based on hysteroscopic findings including amount of cavity involved and type of adhesion as well as clinical symptoms (menstrual status) according to the American Society of Reproductive Medicine (ASRM) scoring system (10). After hysteroscopic adhesiolysis, for both groups, a Foley catheter was inserted into the uterine cavity. Two days after hysteroscopy, the Foley catheter was removed, and 1 ml of PRP was injected into the uterine cavity in women in the PRP group, but no PRP injection was given to women in the control group. To evaluate IUAs by the ASRM score, all cases and controls underwent diagnostic hysteroscopy for 8 to 10 weeks following the intervention (Figure 1).

Menstrual bleeding duration in the case and control groups was assessed by questionnaire.

PRP preparation method: In the PRP group, on the 13${}^{\mathrm{th}}$ day of the menstrual cycle, 8.5 ml of peripheral venous blood was taken through a syringe containing 1.5 ml of acid citrate, as well as an anticoagulant solution (ACD-A) (Arya Mabna Tashkhis, Iran); it was then centrifuged immediately at 1600 g for 10 min. Division of the blood into three layers was performed: cellular plasma situated over, red blood cells (RBCs) at the bottom, and a buffy coat layer in between the two other layers. The buffy coat and plasma layer were both collected and transferred to another tube so as to be centrifuged again at 3500 g for 5 min; 1.5 ml PRP at a 4-5 times higher concentration and 2000 lymphocytes were finally obtained.

### Ethical consideration

Data collection and document review in this study were in accordance with the Ethics Committee of the Research and Clinical Center for Infertility, Shahid Sadoughi University of Medical Sciences, Yazd, Iran (Code: IR.SSU.MEDICINE.REC.1396.256), and with the 1964 Declaration of Helsinki and its later amendments or comparable ethical standards. All study participants provided written informed consent.

### Statistical analysis

Data were analyzed using Statistical Package for the Social Sciences 20.0 (SPSS, SPSS Inc, Chicago, Illinois). Continuous data were presented as the mean ± standard deviation and were assessed by independent Student's t test. P-values < 0.05 were considered statistically significant.

## 3. Results

**Figure 2:**
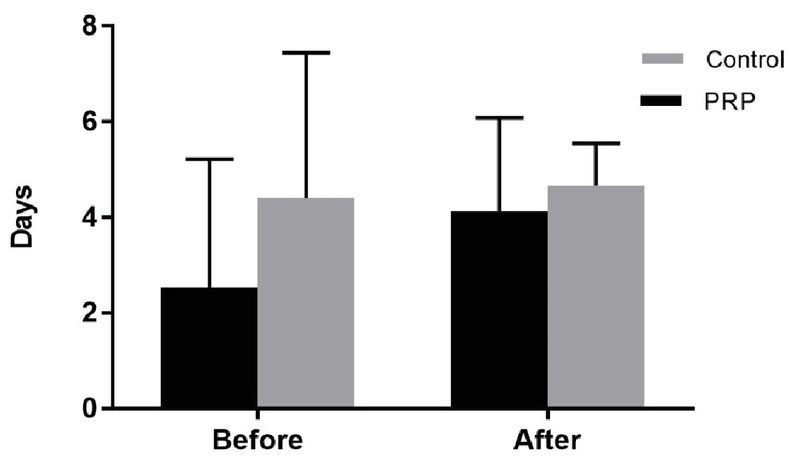
Menstrual bleeding duration (days) of both groups before and after treatment.

The demographic characteristics of the study patients were similar in both groups (Table I). Our results did not reveal any significant difference in the menstrual pattern of women in both groups before and after treatment (Table II). The IUA stage of both groups before and after treatment was similar (Table III), and the menstrual bleeding duration of both groups before and after treatment was also similar (Figure 2).

**Table 1:** Demographic characteristics of the study patients in the two groups


**Variable**	**PRP group (n = 15)**	**Control group (n = 15)**	**p-value**
**Age (year)**	35.93 ± 8.61	36.53 ± 5.38	0.821*
**Gravidity**			
**Nulligravida**	8 (53.33)	6 (40)	
**Multigravida**	7 (46.66)	9 (60)	1.000**
**Possible etiology of AS**			
**Myomectomy**	6 (40)	2 (13.33)	
**Surgical hysteroscopy**	3 (20)	5 (33.33)	
**D & C**	6 (40)	8 (53.33)	0.248**
Data are presented as the Mean ± Standard Deviation (SD) and number (%); *Student's t test; **Chi-squared test; PRP: Platelet-rich plasma; AS: Asherman's syndrome; D & C: Dilation and curettage			

**Table 2:** Menstrual pattern of both groups before and after treatment


**Variable**	**PRP group (n = 15)**	**Control group (n = 15)**	p-value*
**Before treatment**			
**Amenorrhea**	2 (13.33)	0	
**Hypomenorrhea**	10 (66.66)	9 (60)	
**Normal menstrual bleeding**	3 (20)	6 (40)	0.217
**After treatment**			
**Amenorrhea**	0	0		
**Hypomenorrhea**	6 (40)	6 (40)	
**Normal menstrual bleeding**	9 (60)	9 (60)	0.645
Data are presented as numbers (%); *Chi-squared test; PRP: Platelet-rich plasma			

**Table 3:** Intrauterine adhesion stage of both groups before and after treatment


**Variable**	**PRP group (n = 15)**	**Control group (n = 15)**	p-value*
**Before treatment**			
**Stage I**	2 (13.33)	1 (6.66)	
**Stage II**	2 (13.33)	4 (26.66)	
**Stage III**	11 (73.33)	10 (66.66)	0.592
**After treatment**			
**Stage I**	2 (13.33)	6 (40)		
**Stage II**	10 (66.66)	6 (40)	
**Stage III**	3 (20.01)	3 (20)	0.223
Data are presented as numbers (%); *Chi-squared test; PRP: Platelet-rich plasma; Stage I: Mild; Stage II: Moderate; Stage III: Severe			

## 4. Discussion 

The objective of this study was to examine the potential intrauterine employment of PRP so as to improve processes that are associated with endometrial regeneration needed for the management of AS. However, we did not show that the intrauterine injection of PRP improves hysteroscopy outcomes after surgical removal of IUAs. These findings fail to be in agreement with those of a previous study conducted on a small sample, which recognized that the development of postoperative IUAs is lowered by PRP, as evaluated by subsequent hysteroscopy.

Studies have recently worked on murine models and identified the potential of BMDSCs in improving the regeneration of damaged endometrium (11).

However, there is still controversy in terms of the usability and safety of BMDSCs. Researchers tend to know more about their immunogenic reactions and their impact on endometrial proliferative disorders, including infections, endometriosis, as well as the invasive process of harvesting these cells (12).

In Jichun Tan and coworker study that performed on seven patients suffered from AS, first, autologous menstrual blood-derived stromal cells (MenSCs) were isolated and cultured from the menstrual blood of women within ∼2 weeks; they were then transplanted back into women's uterus. Following cell therapy, endometrial growth and pregnancy were appraised the results of which indicated a signiﬁcant improvement in ET (13). Yet, in another study, S. Zhang *et al*. employed transplantation of MenSCs and treatment with PRP to reveal the great capacity of MenSCs in cell-based treatment of AS. Endometrial proliferation and vascular numbers escalated following transplantation; however, fibrosis hyperplasia was improved. Moreover, PRP increased the stemness properties of MenSCs and heightened their effectiveness in vivo, and, specifically, inhibition of inflammatory effect was revealed. Accordingly, the promising capacity of PRP in cell-based therapy for AS has been confirmed and is being considered as potentially vital for clinical application (14).

In women who underwent frozen ET cycles for the first time, Chang *et al*. evaluated the role of autologous PRP in the thin endometrium in five patients and demonstrated an increase of endometrial thickness at 48 to 72 h after PRP infusion in all patients (>7 mm on the day of progesterone administration) so that all of them became pregnant (6).

In another study, we appraised the impact of PRP in women with thin endometrium during a frozen-thawed ET cycle. It was a randomized clinical trial in which 83 women suffering from a poor endometrial response to standard hormone replacement therapy (HRT) were assessed. Whereas both groups (control and case) received HRT, the PRP group was infused by additional 0.5-1 cc of PRP into the uterine cavity on the 13th day of the HRT cycle, the result of which exhibited a significant increase in the endometrial thickness in the PRP group; in this group the implantation rate and per-cycle clinical pregnancy rate were of significant rise as well (15).

The first cases of intrauterine PRP infusion for AS treatment were presented by L. Aghajanova. The treatment was well tolerated, as it showed no short-term or long-term complications, and seemingly improved endometrial function, as demonstrated by successful conception and ongoing clinical pregnancies (16).

In a study of 60 patients with a history of primary or secondary infertility with severe IUAs, who also wished to conceive, Amer *et al*. evaluated the effect of intrauterine injection of PRP following hysteroscopy. Thirty patients (case group) were injected with PRP and 30 others (control group) with IU balloon, the results of which revealed a significant increase in menses duration among the PRP group after surgery (3.0 ± 1.2 days) and an increase in preoperative menses duration (1.4 ± 1.5 days) compared with the balloon group after surgery (1.8 ± 1.3) and preoperative (1.3 ± 1.4) days (17). In that study, which differed from ours, a higher volume of PRP was injected, and the injection of PRP was performed immediately after hysteroscopy; we, however, injected PRP postoperatively with a 48-h delay.

In an experimental model of ethanol-induced damage, Jang *et al*. demonstrated possible recovery using autologous PRP treatment to improve endometrial regeneration. They concluded that intrauterine administration of autologous PRP in a murine model of damaged endometrium stimulates and accelerates the regeneration of the endometrium and lowers fibrosis (9).

Moreover, the evaluation of the effect of PRP on different human endometrial cells involved in tissue regeneration was performed by Lusine and colleagues. They asserted that autologous PRP can induce endometrial regeneration (18).

Because of our concern about dilution of a low volume of PRP in the remaining fluid within the uterus after hysteroscopy, we injected PRP two days after hysteroscopy, whereas in most other studies, PRP was injected immediately after hysteroscopic surgery. It appears that injection immediately after surgery leads to better outcomes.

In IUA surgery, the ultimate goal is to improve pregnancy outcomes and not to restore a normal uterine cavity or normal menstruation. In the present study, no attempt was made to evaluate the pregnancy rate following PRP application for the treatment of IUAs with follow-up for pregnancy outcomes.

## 5. Conclusion

Our findings showed that PRP cannot change the menstrual pattern or the development of postsurgical AS, as evaluated by follow-up hysteroscopy.

##  Conflict of Interest

The authors have no financial or nonfinancial conflicts of interest.
