# Correction to “Platelet-rich plasma in the management of Asherman’s syndrome: An RCT” [Int J Reprod BioMed 2020; 18: 113-12]

**DOI:** 10.18502/ijrm.v19i4.9068

**Published:** 2021-04-22

**Authors:** Atiyeh Javaheri, Katayoon Kianfar, Soheila Pourmasumi, Maryam Eftekhar

The publisher has been informed of an error that occurred on pages 113-120 (Vol. 18; No. 2) which the study type in the title must be changed to “non-randomized clinical trial”. The publisher wishes to apologize for this error. The online version of the article has been updated on April 16, 2021 and can be found at http://journals.ssu.ac.ir/ijrmnew/ article-1-1422-en.html (doi.org/10.18502/ijrm.v18i2.6423).

